# Antidotes to Plague

**Published:** 1900-11-03

**Authors:** 


### ANTIDOTES TO PLAGUE.

At the present moment, when we may any day find ourselves face to face with the problem of dealing with an outbreak of plague, it is well that we should have clear ideas concerning the methods which are in vogue for preventing and, if possible, for curing the disease by specific as distinct from general measures.

Naturally all hygienic and general sanitary means must be brought into operation—notification, isolation, removal to hospital, cleansing and disinfection of the dwelling, flushing and disinfection of the drains, careful scavenging, &c.—and as regards the patient all those measures which general medicine and general surgery suggest. These are things which we should do in attempting to control any infective disease which was threatening to take on epidemic form, and with the details of carrying out such proceeding, we are all familiar enough. But in addition to such means we have in regard to plague certain specific measures which have for their object the production of immunity to the infection; and as very few practitioners can have any practical acquaintance with these proceedings, all information on the subject is of much interest at the present time. Dr. Balfour Stewart,[1] of the Thompson-Yates Laboratories, Liverpool, dealing with this subject, first takes up the general question of how the human or animal body may be rendered immune to an infectious disease.

### Artificial Immunity.

It has long been known, he says, that immunity to the virulent disease is in general obtained by passing through a modified form of it, but the first experiments in this direction with artificial cultures were made in 1880 by Pasteur, who showed that immunity might be induced by inoculation with attenuated virus; that is, with cultures of attenuated but living microbes. Then in 1884 Ferran protected guinea-pigs against cholera in the same way; and in 1893–4 Haffkine started protective inoculations against cholera in man on a large scale in India.

But the method of inoculating with living microbes has obvious disadvantages when carried out with the germ of a disease which may take on a septicæmic form, and this led Haffkine, when he came to experiment with plague, to try to immunise animals by inoculating them with microbes which had been killed by heat. That this was possible in regard to cholera and other disease had already been observed both by himself and others.

### The Immunising Substance.

As to the manner in which protection is obtained by any of these means, it may be said that when an animal is inoculated with either the attenuated living or with the dead microbe it undergoes a reaction which lasts several days, during which the tissue cells of the animal, being stimulated by the specific substance contained in the bodies of the microbes, are induced to manufacture an antitoxin or immunising substance. Thus the animal acquires an active immunity.

Immunity may also be obtained by injecting the serum of an animal which has been forced by repeated inoculations with the virus to produce a supply of this immunising material. But "an animal that has been at the trouble of manufacturing its own antitoxin has a more lasting and efficient protection than one that has been injected with an antitoxic serum obtained secondhand, as it were, from an immunised animal." The antitoxic serum confers immunity almost directly, and thus, as in diphtheria, may be used in the treatment of the disease after it has developed as well as for prophylactic purposes, but its effects are fleeting; a fact which one would expect—considering that the serum is a foreign body and is soon got rid of.

### The Three Methods.

We may say, then, that, putting on one side Lustig's vaccine and serum, the utility of which seems at present somewhat doubtful, there are three methods by which immunity to an infection may be obtained. First, by the introduction of living but modified germs—vaccination; second, by introducing prophylactic fluids such as those devised by Haffkine, in which the microbes have been killed by heat. The action of both of these is to stimulate the tissues to produce immunising substances. Third, by the injection of these immunising substances ready made contained in the serum derived from immunised animals.

### Haffkine's Prophylactic.

In regard to plague we have only to consider the two latter, namely, Haffkine's prophylactic fluid and Yersin's serum. Haffkine's fluid contains both the dead microbes and the broth in which they have grown. It is the intracellular poisons of the dead microbes that are mostly concerned in conferring protection, the object of introducing the broth as well being apparently to accustom the tissues to the poison given out by the microbes in the cultivation medium. "This theory assumes that the intracellular poisons induce a bactericidal power in the tissues, and that the extracellular poisons induce antitoxic properties which would come into play suppose the bactericidal power were insufficient to prevent infection."

Dr. Balfour Stewart states that he has shown that even by itself the broth in which the microbes have grown, apart from the bodies of the microbes, is of use as a means of protection. It must be definitely understood, on the one hand, Haffkine's prophylactic is not a serum, and on the other that it does not, as has sometimes been asserted, induce a mild attack of plague. No living germ is injected, and no self-multiplying disease is set up. Haffkine's fluid is in fact a sterilised culture of the plague microbe in broth. In India, where the prejudices of Hindu and Mohammedan have to be considered, the broth employed is made from goat's flesh; but ordinary broth, as usually made in the laboratory, is really better for the purpose.

### The Process of Inoculation.

As to the method of using this fluid Dr. Margaret Christie, late plague medical officer in Calcutta, who has inoculated a large number of persons, says that the dose varied from 5 c.cm. to 20 c.cm., the ordinary dose of the fluid provided while she was doing this work being 5 c.cm. Roux's syringes, with a capacity of 10 c.cm., were employed. To save time the whole of the syringe was filled and used to inoculate several people, the graduation on the cylinder making it possible to gauge the dose accurately for each person. The needle was well rubbed with a cotton-wool sponge dipped in 1 in 20 carbolic lotion in the interval between two inoculations. Strict aseptic precautions were observed throughout. "Carbolic acid was used because it has been proved to have no action upon the prophylactic. The neck of the sealed bottle of prophylactic was sterilised in the flame of a spirit lamp. The cork was then extracted with sterilised forceps. The bottle was covered with a pad of absorbent cotton just squeezed out of carbolic lotion until the syringe was quite ready, and in filling the syringe the bottle was held on the slant. The syringe was carefully taken to pieces each day, after it had been used, and washed with boiling water, then with carbolic, 1 in 20, and carefully dried. Just before using the needle it was boiled in a test tube and inserted into the syringe with sterilised forceps."

Dr. Christie invariably selected the back and outer side of the arm, midway between the shoulder and elbow, as the site of inoculation, but in mens he believes the flank was sometimes used. The skin was well rubbed with a sponge dipped in 1 in 20 carbolic, the needle was run into the superficial fascia for quite half or three-quarters of an inch, and the fluid slowly injected; the needle was then quickly withdrawn, the skin being pinched up as it emerged. If the needle were run in towards the elbow it quite prevented any of the fluid escaping with it. The patient was allowed to press a small sponge, squeezed out of carbolic lotion on the spot, to prevent any bleeding, but it was not necessary to apply any dressing at all. The height of the fever of reaction varied a good deal, but it rarely lasted more than two days, though the arm remained painful and tender for a week or ten days, and a small hard nodule often remained for a few weeks.

Mr. Haffkine considers it desirable to reinoculate all cases in which the temperature does not rise to 103° F.

### Yersin's Antitoxic Serum.

Turning now to serums the antitoxic serum of Yersin is not only used as a curative agent for plague, but also acts as a prophylactic. But the immunity conferred by it is very short—only about fifteen days. The principle on which the use of this serum is founded is the same as that of an antidiphtherial serum. An animal, preferably a horse, is immunised by repeated and gradually increasing inoculations with either dead or living cultures of the specific microbe, and thus acquires an active immunity. If the serum of such an animal be inoculated in considerable quantities into a human being suffering from this specific disease this is ameliorated or cured. The serum acts in two ways: it may be bactericidal (that is, it may prevent the growth of the microbe in the body) or it may be antitoxic (that is, may neutralise the poisonous products given out by the microbes in growing) or it may be both. As a prophylactic a bactericidal serum will do, but for it to act as a curative agent a serum must have antitoxic properties to counteract the poison already formed by the microbe as well as bactericidal properties to arrest its growth. Now it seems that the antitoxic properties are more marked in serum prepared by injecting living cultures than in those made by injecting cultures that have been killed. There are some difficulties in the way of using the living cultures owing to their local action on the tissues of the animal, but latterly the Pasteur Institute has adopted the plan of partially immunising the horses with cultures killed by heat and then going on with living cultures, and the results are said to be more satisfactory than those obtained by the use of the dead ones throughout. The manner of using the serum is practically the same as that with which we are familiar in regard to the antidiphtheritic serum now so generally employed against in the treatment of diphtheria.

### The Methods Contrasted.

The point which we have to remember in regard to the use of these two products is that while Haffkine's fluid is intended for prophylactic purposes alone, giving an immunity which although not perhaps complete may last for a considerable time, and thus may be found very useful for those who have from the nature of their duties to be exposed to the infection of the disease, Yersin's serum, on the other hand, gives an immediate immunity considerable in degree, but evanescent, so that its chief utility is the treatment of the disease, in the protection of "contacts"—people who are believed to have been recently exposed to infection—and in giving temporary protection to those who may have to undertake some especially risky work such as disinfecting an infected ship or clearing out a plague-stricken area.

[1] British Medical Journal, October 27.

